# Detection of Fumonisin B1 and Ochratoxin A in Grain Products Using Microsphere-Based Fluid Array Immunoassays

**DOI:** 10.3390/toxins2020297

**Published:** 2010-02-25

**Authors:** George P. Anderson, Vasudha A. Kowtha, Chris R. Taitt

**Affiliations:** Center for Bio/Molecular Science & Engineering, Naval Research Laboratory, Washington DC, 20375, USA; Email: george.anderson@nrl.navy.mil (G.P.A.)

**Keywords:** mycotoxin, immunoassay, bead array, fumonisin, ochratoxin, detection

## Abstract

Grain products are a staple of diets worldwide and therefore, the ability to accurately and efficiently detect foodborne contaminants such as mycotoxins is of importance to everyone. Here we describe an indirect competitive fluid array fluoroimmunoassay to quantify the mycotoxins, fumonisin B1 and ochratoxin A. Both toxins were immobilized to the surface of microspheres using a variety of intermediate molecules and binding of biotinylated "tracer" antibody tracers determined through flow cytometry using streptavidin-phycoerythrin conjugates and the Luminex100 flow cytometer. Competitive assays were developed where the binding of biotinylated monoclonal antibodies to fumonisin B and ochratoxin A was competitively inhibited by different concentrations of those toxins in solution. Concentrations of fumonisin giving 50% inhibition were 300 pg/mL in buffer, 100 ng/g in spiked oats, and 1 μg/g in spiked cornmeal; analogous concentrations for ochratoxin A were 30 ng/mL in buffer, 30 ng/g in spiked oats, and 10 ng/g in spiked corn. The future challenge will be to expand the number of mycotoxins tested both individually and in multiplexed format using this platform.

## 1. Introduction

Mycotoxins are toxic secondary metabolites secreted by fungi into the environment. Fumonisin B1 (FB1) is produced by *Fusarium* molds and ochratoxin A (OTA) is produced by the *Penicillium* and *Aspergillus* molds. These mycotoxins are found mainly in grain products (e.g., oats, corn, wheat); however OTA is also found in pork products, as well as coffee [1], wine grapes [2] and dried grapes. Mycotoxins pose crucial agricultural and health concerns, and are responsible for billions of dollars in economic losses each year. Both OTA and FB1 are known nephrotoxins, hepatotoxins, and potential carcinogens, and have been associated with reproductive toxicity, including neural tube defects. In contrast to bacterial, viral, and many toxic foodborne contaminants, mycotoxins are not inactivated by extreme temperatures; therefore, monitoring of cereals and other affected food products *prior* to distribution is vital. For a comprehensive review of mycotoxin properties, ecology, and monitoring efforts, see Cousin *et al.* [3].

Mycotoxins are toxic in trace amounts, therefore assays must be extremely sensitive. Additional qualities desired in an efficient and accurate mycotoxin detection technique include a minimum of sample preparation and cleanup steps, low cost, minimal dependence upon extensively trained personnel, and rapid time-to-result. 

Rapid immunoassays have been developed for a number of mycotoxins [4,5,6,7,8,9]. Many of these have utilized a competitive assay format involving the competition between the target antigen in the sample and an immobilized antigen (or analog) for binding to a labeled antibody. The amount of mycotoxin in the sample is then quantified by the decrease in antibody binding to the detection surface; therefore, the signal measured changes inversely with the amount of the target antigen in the sample. 

Quantitative fluorescence cytometry is an efficient technique to rapidly examine large groups of analytes for multiple antigens and binding sites at once. Luminex 100, a specialized flow cytometer, can perform multiplexed assays by differentiating up to 100 fluorescent microspheres (bead sets). These bead sets are identified by having two dyes incorporated within each bead at one of ten different concentrations (each) to form a 10 × 10 array; these dyes are excited by the system's red laser, and by using the intensity of fluorescence from the two dyes, the instrument is capable of determining which bead is present. In this manner, large numbers of different bead sets can be combined together to create customizable "bead arrays" for multiplexed detection. The system utilizes a green laser to quantify the tracer fluorophore that is distinct from the coding dyes and indicates the immunoassay signal on each individual bead. As the flow cytometer identifies and quantifies the tracer fluorescence for each microsphere sequentially, it facilitates testing in a multiplexed format, and allows rapid evaluation of antibodies and assay conditions. We have previously utilized this instrument to develop competitive immunoassays for the explosive TNT [10,11]. 

In this work, Luminex 100 microspheres were coated with FB1 and OTA attached covalently through various intervening molecules. The toxin-coated beads were incubated with a mixture of FB1, OTA, and biotinylated anti-FB1 and anti-OTA "tracer" antibodies, in a competitive immunoassay format; streptavidin-phycoerythrin conjugate was then used to quantify the amount of tracer antibody bound to the beads. Grain samples spiked with FB1 and OTA to represent naturally contaminated samples were also analyzed.

## 2. Results and Discussion

### 2.1. Optimization of assay conditions

**Figure 1:**
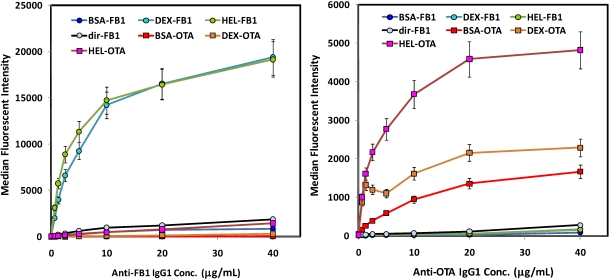
Direct binding assays of antibodies to mycotoxin-decorated beads (no competition). Left: FB1 assays using biotinylated anti-FB1 IgG tracer; FB1-coated beads are shown with circle symbols, whereas OTA-coated beads are shown with square symbols. Right: OTA assays using biotinylated anti-OTA IgG. Beads derivatized with mycotoxins are indicated as follows: FB1 *via* its amine (dir-FB1); FB1 *via* its carboxyls through BSA (BSA-FB1), lysozyme (HEL-FB1), or aminodextran (DEX-FB1); OTA *via* its carboxyl through BSA (BSA-OTA), lysozyme (HEL-OTA), or aminodextran (DEX-OTA).

Initial experiments were designed to evaluate binding of the anti-toxin antibodies to the various toxin-coated microspheres before a competitive assay was developed; dose-response curves were generated to assess the optimal concentration of tracer antibodies that provide both strong signal in the absence of toxin, but whose binding to the toxin-coated beads could be effectively competed at low concentrations of soluble mycotoxin. While K_d_s have not been determined for either antibody, neither approached binding saturation rapidly, indicating fairly low affinities. Thus, one is forced to compromise between using sufficient antibody to generate a strong signal, while limiting excess antibody which would negatively affect assay sensitivity. We chose concentrations of anti-FB1 and anti-OTA in the Luminex assays in the low μg/mL range (Figure 1). A higher concentration of anti-OTA than anti-FB1 was required to generate a robust fluorescent signal, which may in part explain why in buffer the sensitivity of OTA assays was lower than that of FB1 assays. 

**Figure 2:**
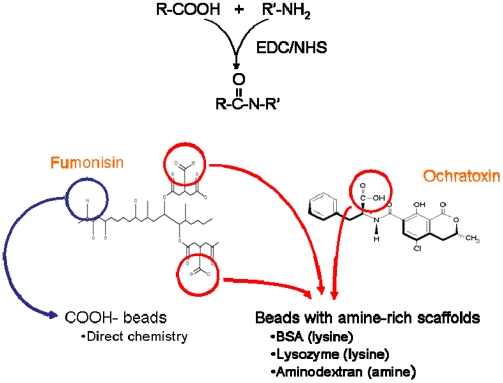
Overall scheme for attachment of mycotoxins to microspheres. Top: EDC-mediated coupling of carboxyls to amines. Lower left: Fumonisin can be attached to carboxy-decorated beads *via* its single amine (blue arrow) or to beads decorated with amine-rich scaffolds *via* its two carboxyl residues (red). Lower right: Ochratoxin is attached to amine-decorated beads *via* its carboxyl moiety.

As mycotoxins and other small molecules most often possess only a single epitope and are frequently bound by antibodies in only one orientation, we addressed the critical issue of antigen presentation on the beads. Since the beads are commercially available with carboxyl-modified surfaces, FB1 can be linked directly to carboxyl-microspheres through its available amine using EDC chemistry (Figure 2, left side, blue arrow). However, OTA does not possess a primary amine for EDC-mediated linking, but rather, a carboxyl residue; FB1 additionally possesses two carboxyl moieties. To allow immobilization of the toxins *via* their carboxyl groups, we converted carboxyl-decorated microspheres to amine-decorated ones using a number of amine-rich scaffolds. These large molecules have the additional benefit of potentially increasing both the number and density of amines available for toxin attachment. BSA was used to provide a surface with low non-specific binding, whereas both lysozyme and amino-dextran were used to provide surfaces with a preponderance of amines available for the next reaction (Figure 2, "beads with amine-rich scaffolds").

Direct binding studies of biotinylated antibodies to these various toxin-coated microspheres showed a similar pattern for both the OTA and the FB1 (Figure 1): highest binding was obtained on both OTA and FB1 beads where the mycotoxins were immobilized through lysozyme and aminodextran. Interestingly, anti-FB1 antibodies bound very poorly to microspheres on which FB1 had been immobilized *via* its primary amine (Figure 1, left panel; light blue circles, black line). These results imply that the amine on FB1 is a critical part of the anti-FB1 antibody binding epitope. However, as EDC-mediated coupling results in a zero-length link, it is possible the signal was also reduced by limited freedom of movement of the directly-immobilized FB1; further immobilization studies utilizing linkers directed towards this end of the FB1 molecule should help elucidate the mechanism. In general, these results underscore the value in attaching a target, especially small molecule targets, to the surface using a variety of linkers that can provide a range of flexibilities and densities.

### 2.2. Mycotoxin assays in buffer

**Figure 3:**
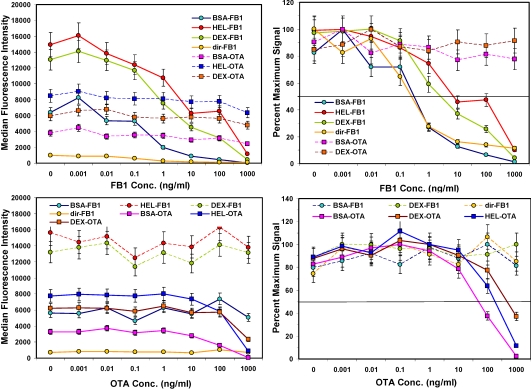
Competitive assays in buffer. FB1 or OTA were spiked into PBST, mixed with toxin-coated beads, and then incubated with a mix of both biotinylated-Mab tracers. After binding, unbound antibodies were removed by filtration and SA-PE was added prior to measurement on the Luminex 100. Top: FB1 assays; FB1-coated beads are shown with solid lines, whereas OTA-coated beads are shown with dashed lines. Bottom: OTA assays; OTA-coated beads are shown with solid lines, whereas FB1-coated beads are shown with dashed lines. Error bars represent SD.

After the concentration of biotinylated tracer antibody was selected, the ability to inhibit the signal generated was examined by testing buffer samples with toxin spiked in at a wide range of concentrations. As expected, the higher the concentration of toxin, the lower the signal measured (Figure 3, left hand panels). Signals from non-specific bead sets (*i.e.*, coated with a different toxin) were high and did not drop significantly. These results indicated that specificity of the bead sets was as expected: OTA present in solution caused inhibition on only OTA-coated beads and not FB1-coated beads, while FB1 in solution affected only FB1-coated beads and not OTA-coated beads. Although the microspheres with fumonisin immobilized *via* its available amine were not bound well by the anti-FB1 antibody (solid orange line, upper left panel), when displayed as percent maximum signal (upper right panel), the inhibition curve was remarkably similar to that where FB1 was immobilized by its carboxyls using BSA as a scaffold.

### 2.3. Mycotoxin assays in food products

Various food extracts have been shown to affect mycotoxin immunoassays [3,4,12,13,14]. To distinguish between interference from the sample matrix and overall extraction efficiencies, our approach was to test spiked control extracts, and later apply to grain samples spiked *before* extraction. Since most validated methods for mycotoxin extraction from grains involve organic solvents, our initial experiments utilized methanolic extracts made from unspiked ground corn and oats. In contrast to our previous studies [7,8,9,15], even the presence of low methanol concentrations (5%) produced inconsistent results. We therefore tried drying down the methanolic extracts under nitrogen and resuspending them in buffer before analysis. Assay results from the dried methanolic oat extracts were reproducible and relatively sensitive; 10 ng/g concentrations of both FB1 and OTA gave 50% inhibition relative to controls. However, methanolic corn extracts showed an increase in signals from FB1-coated beads at low FB1 concentrations and high concentrations of OTA gave rise to (non-specific) inhibition on FB1-coated beads in the corn extracts (data not shown). When combined with the significant increase in overall processing time due to methanol removal, we chose to test extraction of mycotoxins from the corn and oatmeal in an aqueous buffer; this method has been shown effective in corn [16]. Other methods with potential to mitigate this effect include solvent partitioning/removal [17,18] and use of recognition molecules tolerant to high methanol [19,20]. 

**Figure 4:**
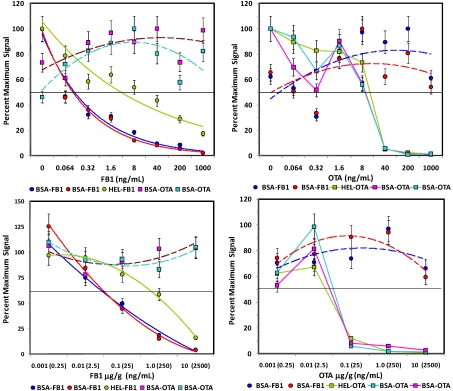
Competitive assays of spiked oats. Upper panels: Assays of control extracts spiked with FB1 (left) or OTA (right). Lower panels: Assays of oats spiked with FB1 (left) or OTA (right) *before* extraction. Plots are shown as percent of the maximum (uninhibited) signal. Microsphere sets specific for the FB1 are shown as square symbols, OTA-coated beads are shown as circle symbols. Error bars represent SEM.

The sensitivity for the toxins spiked into the aqueous oat extracts (Figure 4, top panels) was generally similar to that observed in buffer (Figure 3), although the response of the non-specific bead sets became more erratic (dashed lines), especially in OTA assays. These results indicate that a large degree of inhibition must be observed on the toxin-specific beads (e.g., 50% inhibition) to attribute any inhibition to the presence of the toxin with confidence. In contrast to oats extracted with methanol, the PBST oat extract was quite viscous, making filtration on the microtitre plate difficult. Future work in this matrix could potentially utilize MagPlex beads to facilitate separation and washing steps.

**Figure 5:**
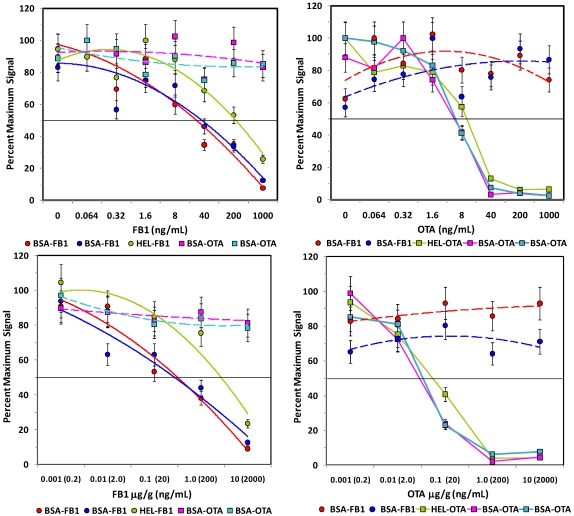
Competitive assays of spiked corn. Upper panels: Assays of control extracts spiked with FB1 (left) or OTA (right). Lower panels: Assays of corn spiked with FB1 (left) or OTA [right] *before* extraction. Plots displayed are analogous to those in Figure 4.

Analogous experiments were performed with corn samples spiked before and after extraction with aqueous buffer (Figure 5). In contrast to the methanolic extracts, signals were more reproducible in the aqueous extracts with no artifacts. Although response in OTA assays was similar to that observed in both buffer and oat extract, inhibition in FB1-spiked corn extracts was dampened; 50% inhibition was observed at 40 ng/mL or higher in spiked corn extract, whereas this same inhibition was observed at a concentration at least 4-fold lower in buffer and oat extracts.

After the extent of interference due to the oat and corn matrices was evaluated, FB1 and OTA were spiked into the ground grains *prior* to extraction to determine the extent of sample loss due to the extraction procedure (Figure 4 and Figure 5, lower panels). In contrast to studies using dried methanol extracts, FB1 and OTA could be detected in aqueous extracts of corn, as well as oats. However, while over 50% recovery was obtained when using methanol to extract FB1- and OTA-spiked oats, 1.5% and 15% recoveries was obtained from FB1- and OTA-spiked oat samples, respectively, when extracted with PBST. The exceedingly low recoveries may well be due to the viscosity of the PBST oat extract or to the presence of colloidal particulates, even when spun immediately before analysis. On the other hand, 20-40% recovery was obtained with PBST extracts of corn. Overall, these results were not surprising, since methanolic extractions have been demonstrated to have higher efficiencies than fully aqueous extractions [16]. 

In spite of extraction efficiencies of <50%, FB1 was detectable at levels below FDA Advisory Levels (Table 1) and gave a fairly linear response over a large concentration range. Thus, the FB1 assays were predictive of toxin concentration in both corn and oats. OTA assays, on the other hand, exhibited a very sharp transition in its dose-response, indicating that quantification for that toxin would be difficult in this matrix. However, at OTA concentrations over 100 ng/g, a yes/no determination could be made under the present assay conditions. Clearly however, significant improvement must be obtained for detection of OTA to assure contamination is below the required level (2 ng/g).

**Table 1:** Mycotoxin concentrations giving 50% inhibition (BSA-FB1- or BSA-OTA-coated beads).

	Buffer	Oats		Corn		FDA Level
		Spiked extract	Spiked before extraction	Spiked extract	Spiked before extraction	
FB1	~0.3 ng/mL	0.32 ng/mL	100 ng/g	40 ng/mL	1 μg/g	2 ppm
OTA	~30 ng/mL	0.8 ng/mL	~30 ng/g	8 ng/mL	100 ng/g	5 ppb

Overall, this work has demonstrated that competitive fluid array immunoassays can readily be developed for detection of mycotoxins found in food products. Although both FB1 and OTA could be detected in spiked oats and corn, sensitivity was a serious issue for OTA; FDA advisory levels for OTA are in the ppb, rather than ppm, range. Although initial experiments using methanol extraction protocol proved problematic with corn samples, a simple extraction using PBST could be used to detect FB1 in both corn and oats. Although the loss in extraction efficiency was a significant limitation, the PBST extraction eliminated the time-consuming drying step and provided sample extracts in a buffer compatible with Luminex immunoassays. Nonetheless, sample preparation is clearly a major consideration, and may require optimization for each food matrix being tested. There are a number of commercially available rapid sample preparation and/or solvent clean-up kits that can be used to accomplish the same purpose [21,22]. Although the Luminex 100 system can evaluate up to 100 different sets of microspheres simultaneously, for most of our experiments we only utilized 6 sets at a time to assay for two different mycotoxins in triplicate. A number of attempts were made to develop the assay as a simple homogeneous, one-step assay where all the reagents (microspheres, antibody, SA-PE, and sample) were simply mixed and measured (data not shown). While the homogeneous assays performed well in spiked buffer samples, tests in the food matrices were disappointingly erratic. After several failed attempts, the homogeneous method was abandoned in favor of the two-step method described here (incubation of sample/microspheres/antibody, followed by incubation with SA-PE conjugate). The two-step method, while requiring more handling steps, alleviates the need to accurately balance the amount of biotinylated antibody and SA-PE, a process which would become increasingly more problematic as one attempts to increase the level of multiplexing. That being said, we anticipate that the ability to multiplex mycotoxins immunoassays will likely be limited more by immuno-reagent capability than by the analytical system. 

## 3. Experimental Section

### 3.1. Buffers and reagents

Unless otherwise specified, all chemicals were used as received. Standard carboxy-functionalized xMAP microspheres were purchased from Luminex Corp. (Austin, TX). N-Hydroxysulfosuccinimide (sulfo-NHS), 1-ethyl-3-(3-dimethylaminopropyl)carbodiimide (EDC), and NHS-LC-LC-biotin were purchased from Pierce (Rockford, IL). Fumonisin B1 (F1147), ochratoxin A (O1877), bovine serum albumin (BSA), hen egg lysozyme, aminodextran (70 kDa, 32 amines per dextran), anti-OTA IgG1 (Mab 5E2) and anti-FB1 IgG1 (Mab 1D6) were purchased from Soft Flow Biotechnology (St. Louis Park, MN), and streptavidin-phycoerythrin conjugates (SA-PE) were kindly provided by Prozyme (San Leandro, CA). Organic oats and organic corn flakes were purchased from local grocery stores. All other chemicals were of reagent grade.

### 3.2. Covalent binding of FB1 and OTA to microspheres

#### 3.2.1. Preparation of xMAP microspheres with covalently attached linker-linking of amines to COOH-functionalized beads

For these experiments, multiple different microsphere sets were prepared in parallel at the same time. Briefly, 100 μL of each set (1.5 × 10^6^ microspheres) were placed in eppendorf tubes and centrifuged at 14,000 rpm for 4 min. The supernatant was transferred from the primary tubes to “chase tubes” used to minimize the loss of microspheres. 100 μL of 0.1 M Na phosphate buffer, pH 6.0 (Activation Buffer) was added to the primary tubes. Both sets of tubes were centrifuged as before. The supernatants of the chase tubes were discarded, and the supernatants from the primary tubes were again transferred to the chase tubes. This process of washing the beads was repeated once more. After a final centrifugation of the chase tubes, the pelleted beads were then resuspended in Activation Buffer (primary tube: 80 μL; chase tubes: 40 μL). 

Since standard Luminex microspheres are supplied functionalized with carboxyl moieties, EDC-mediated coupling was used to attach the targets and scaffolds (Figure 2). EDC is a water-soluble zero-length crosslinker used to conjugate amine and carboxyl functional groups. It reacts with carboxyl groups, temporarily forming a reactive intermediate which binds to available amine groups. Sulfo-NHS has been shown to stabilize EDC reactions and increase the yield of conjugation substantially [23]. EDC (50 mg/mL in dimethyl sulfoxide [DMSO]) and sulfo-NHS (50 mg/mL in H_2_O) were added to the primary tubes (10 μL of each) and 5 μL of each was added to the chase tubes. Tubes were then incubated in the dark for 20 minutes with occasional mixing. The microspheres in the chase tubes were combined with those in the corresponding primary tubes, then centrifuged as described previously. Again using the chase tubes, the microspheres were washed twice with 0.1 mL Activation Buffer and the primary and chase tube contents combined. Phosphate-buffered saline pH 7.4 (PBS, 50 μL) was added to each of the primary tubes which were then vortexed and sonicated to ensure even dispersion of the microspheres. The material to be immobilized was then added and the microspheres incubated overnight in the dark at 4ºC. Immobilized materials included FB1 (10 mg/mL in PBS, 50 μL added) and several different scaffolds: BSA, hen egg lysozyme, and amino dextran (10 mg/mL stock solutions in PBS, 50 μL added to microspheres). Uncoupled material was removed by centrifugation and the microspheres resuspended in Activation Buffer for addition reaction, or PBS containing 0.05% Tween-20 (PBST) for storage if complete.

#### 3.2.2. Covalent attachment of FB1 and OTA to modified xMAP microspheres-linking of carboxyls to amine-decorated microspheres

As both FB1 and OTA possess carboxyl groups, each could be immobilized by EDC conjugation chemistry to microspheres decorated with amine-containing scaffolds (e.g., BSA, lysozyme, and aminodextran, see above). Separately, FB1 and OTA (0.1 mL each of 1 mg/mL in Activation Buffer) were activated by the addition of 20 μL each EDC (50 mg/mL in DMSO) and sulfo-NHS (50 mg/mL in H_2_O). After 20 minutes incubation, the reaction was quenched by the addition of 1 μL undiluted β-mercaptoethanol. Then 20 μL of the activated toxin was added to each set of amine-scaffold-decorated microspheres and the toxin-microsphere mixtures incubated overnight. Unbound mycotoxins were then removed by washing each bead set twice with 200 μL PBST and resuspending them in a final volume of 400 μL PBST. They were stored in a cold room until use.

### 3.3. Biotinylating anti-FB1 and anti-OTA monoclonal antibodies

A ten-fold molar excess of biotin-LC-LC-NHS (dissolved in DMSO) was added to the antibody (500 μL anti-FB1 or anti-OTA at 1 mg/mL) in PBS. After 1 hour of incubation, free biotin was removed by gel filtration and the antibodies quantified by the OD_280_.

### 3.4. Fluid array immunoassays for FB1 and OTA

Serial dilutions of FB1 and OTA were made within the wells of a 1.2 μm multiscreen 96-well filter plate (Millipore, Billerica, MA; 80 μL/well) using PBST containing 1 mg/mL BSA (PBSTB) as diluent. Each toxin-coated bead set was mixed in an eppendorf tube (0.5 μL/well), washed and resuspended in sufficient buffer to add 5.5 μL of the bead mix to each well. Finally 5.5 μL of the appropriately concentrated antibody (or mixture) was added to each well, and the plate was incubated for 30 minutes. After washing with PBSTB, 50 μL of SA-PE diluted into PBSTB to a final concentration of 5 μg/mL final was added to each well and the plate was incubated for 30 minutes. After a final wash, the microspheres were resuspended in 90 μL PBST and transferred to a standard microtiter plate for measurement by the Luminex 100. Oats and corn meal extracts were tested in a similar manner. For the data analysis of the spiked extracts the response of each bead set was standardized by the response of the HEL-OTA for the FB1 assays or by the HEL-FB1 response for the OTA assays. Using an internal control in each data point helped decrease variability to some extent. 

### 3.5. Preparation and extraction of food samples

Organic oats and corn were ground separately and were weighed out in 2 g aliquots. Each aliquot was spiked with 2 mL of FB1 or OTA solution (2 ng/mL to 20 μg/mL stock solutions, dissolved in methanol), mixed for 2 min on a vortex, and dried overnight in a hood; final concentrations ranged from 1 ng/g to 10 μg/g for each mycotoxin. Unspiked methanol was used as a control. The following morning, each aliquot was incubated with 5 volumes of PBS for 2 hours, centrifuged for 15 min at 5000× g, and the supernate collected; pellets were discarded as hazardous material. Immediately prior to analysis, extracts were centrifuged to remove any residual particulates. To discriminate between the effects of the matrix itself and extraction efficiency, sample extracts were prepared using unspiked oats or corn; after sample extraction, FB1 or OTA was added directly to the extract before analysis.

## 4. Conclusions

The use of the Luminex 100 flow cytometer for detection of two foodborne mycotoxins at sub-ppm levels was demonstrated. Fumonisin B1 could be detected at lower levels than ochratoxin, although more work is required to improve sensitivity for ochratoxin and overall reproducibility before application to real samples in the field. Future work can expand the method to include testing for other grain-contaminating mycotoxins such as aflatoxins, and to increase the number of mycotoxins tested in a multiplexed format; the present study demonstrated simultaneous analysis for only two mycotoxins. Although extraction methods free of organic solvents could be used for detection of FB1 at required levels, improved extraction-combined with increased sensitivity-will be required for detection of OTA in grains at concentrations lower than FDA Advisory levels.
